# Acute lymphoblastic leukemia presenting as myocardial infiltration mimicking hypertrophic cardiomyopathy: a case report

**DOI:** 10.3389/fonc.2026.1910421

**Published:** 2026-07-22

**Authors:** Zhengshuo Jin, Xuehan Mao, Yuehua Huang

**Affiliations:** Department of Hematology, Beijing Tsinghua Changgung Hospital, School of Clinical Medicine, Tsinghua Medicine, Tsinghua University, Beijing, China

**Keywords:** case report, hypertrophic cardiomyopathy, myocardial infiltration, reversible cardiomyopathy, T-cell acute lymphoblastic leukemia

## Abstract

**Background:**

Acute lymphoblastic leukemia (ALL) with myocardial infiltration is a rare extramedullary manifestation, typically encountered in advanced or relapsed disease. Presentation as an isolated, reversible hypertrophic cardiomyopathy (HCM)-like phenotype without typical hematological symptoms is exceptional and poses a major diagnostic challenge.

**Case presentation:**

A 25-year-old man presented with fever, weight loss (20 kg over 3 months), progressive dyspnea, and bilateral lower extremity edema. Echocardiography and CMR imaging revealed severe asymmetrical left ventricular hypertrophy (maximum septal thickness 27 mm) with reduced left ventricular ejection fraction (as low as 27%), initially suggesting non-obstructive HCM. However, the presence of persistent fever, a marked inflammatory response (C-reactive protein 129 mg/L; reference: <10 mg/L), and elevated D-dimer and the lack of a family history or cardiovascular risk factors raised suspicion. Whole-body [^18^F]FDG PET–CT demonstrated intense myocardial uptake (SUVmax 14.8) along with widespread lymphadenopathy and splenic involvement. Peripheral blood smear showed 23% lymphoblasts, and bone marrow examination confirmed T-cell acute lymphoblastic leukemia (T-ALL) with myeloid co-expression. Endomyocardial biopsy revealed diffuse infiltration by T-lymphoblastic cells (CD3^+^, CD5^+^, TdT^+^). The patient received three cycles of chemotherapy (VDPL, venetoclax+chidamide+azacitidine, mitoxantrone+cytarabine). At follow-up after three cycles, bone marrow minimal residual disease was negative, and repeat echocardiography showed normalization of cardiac structure and function.

**Conclusion:**

This case illustrates that T-ALL can first manifest as a rapidly progressive, HCM-mimicking cardiomyopathy that is fully reversible with effective chemotherapy. Clinicians should suspect hematologic malignancy in a young patient with unexplained cardiac hypertrophy associated with constitutional symptoms, elevated inflammatory markers, and poor response to standard therapy for heart failure. Early bone marrow examination and, if necessary, endomyocardial biopsy can confirm the diagnosis.

## Introduction

Acute lymphoblastic leukemia (ALL) is an aggressive hematological malignancy characterized by clonal proliferation of immature lymphocytes in the bone marrow and blood. It typically presents with cytopenias, fever, and organ infiltration (1). Extramedullary involvement commonly occurs in the central nervous system, liver, spleen, and lymph nodes, whereas cardiac involvement is rare in clinical practice (2). However, postmortem autopsy studies have reported cardiac involvement in 20%–44% of leukemia cases (3–5), yet the vast majority of patients with cardiac infiltration remain asymptomatic during life (6). Consequently, antemortem diagnosis of leukemic myocardial infiltration is exceedingly rare.

When clinically evident, cardiac involvement in ALL most commonly manifests as pericardial effusion or cardiac tamponade, but it can also present as an interventricular septal mass, severe myocardial hypertrophy mimicking primary cardiomyopathy, or acute myocardial infarction-like presentations (7–9). Importantly, a small number of ALL patients present with clinical and imaging features indistinguishable from primary hypertrophic cardiomyopathy (HCM), as illustrated by reports of T-cell acute lymphoblastic leukemia (T-ALL) manifesting as isolated cardiac relapse or as biventricular hypertrophy with elevated native T1 and extracellular volume (ECV) values on cardiac magnetic resonance (CMR) parametric mapping (10, 11). These cases demonstrate that leukemic myocardial infiltration can masquerade as HCM, yet the diagnosis is often delayed or missed due to lack of clinical suspicion.

Here, we report a case of a 25-year-old man who presented with fever, weight loss, and progressive heart failure, in whom initial echocardiography and CMR were diagnostic of non−obstructive HCM. The subsequent detection of systemic T−ALL with diffuse myocardial infiltration and complete normalization of cardiac structure and function after chemotherapy highlights a critical diagnostic pitfall and the remarkable reversibility of leukemic cardiomyopathy. This case also underscores the importance of multidisciplinary collaboration between hematologists and cardiologists in managing such patients.

## Case presentation

A 25-year-old man presented to our hospital with a 3-month history of intermittent fever (maximum 38.5°C), unintentional weight loss of 20 kg, progressive dyspnea, and bilateral lower extremity edema over the past month. He had no family history of cardiomyopathy and no cardiovascular risk factors. Initial workup at an outside hospital showed leukocytosis (WBC 10.93 × 10^9^/L), elevated CRP (148.97 mg/L; reference: <10 mg/L), and liver function abnormalities (ALT 144.6 U/L, AST 135.2 U/L, TBIL 51.68 μmol/L, DBIL 31.15 μmol/L). Echocardiography revealed asymmetrical left ventricular hypertrophy with septal thickness of 21 mm and preserved ejection fraction (LVEF 60.4%). Despite empirical antibiotics, fever persisted for 3 weeks and then spontaneously resolved, but dyspnea and edema gradually worsened. One month before admission, repeat echocardiography showed a decline in LVEF to 27% with increased septal thickness up to 25 mm and a hypoechoic mass (13 mm × 13 mm) in the basal segment of the posterior wall. Chest CT demonstrated left pleural effusion with passive atelectasis and mediastinal lymphadenopathy. The patient was treated with left pleural drainage, which temporarily relieved his symptoms, but he continued to lose weight and developed progressive fatigue.

On admission to our hospital, the patient was alert but ill-appearing and emaciated. Vital signs showed a heart rate of 104 bpm, and his blood pressure was normal. Physical examination revealed jugular vein distension, coarse breath sounds with decreased breath sounds on the left, diminished heart sounds without murmurs or friction rub, and bilateral pitting edema of the lower extremities. The abdomen was soft without hepatosplenomegaly.

**Figure 1:**
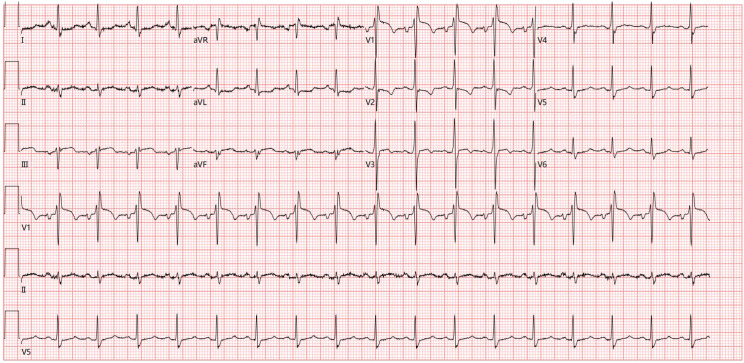
Electrocardiogram on admission. The tracing shows sinus tachycardia (heart rate 104 bpm), left axis deviation, incomplete right bundle branch block, and non-specific T-wave abnormalities in the precordial leads.

Laboratory findings on admission showed a normal white blood cell count (6.8 × 10^9^/L), but CRP remained elevated at 129.68 mg/L. NT-proBNP was 2,103 pg/mL (reference: <300 pg/mL), high-sensitivity troponin I (hsTI) was mildly elevated at 19.4 ng/L (reference: <15 ng/L), and D-dimer was 5.01 mg/L (reference: <0.5 mg/L). Autoimmune markers and thyroid function were normal. Electrocardiogram showed sinus tachycardia, left axis deviation, incomplete right bundle branch block, and non-specific T-wave changes (**Figure 1**).

**Figure 2:**
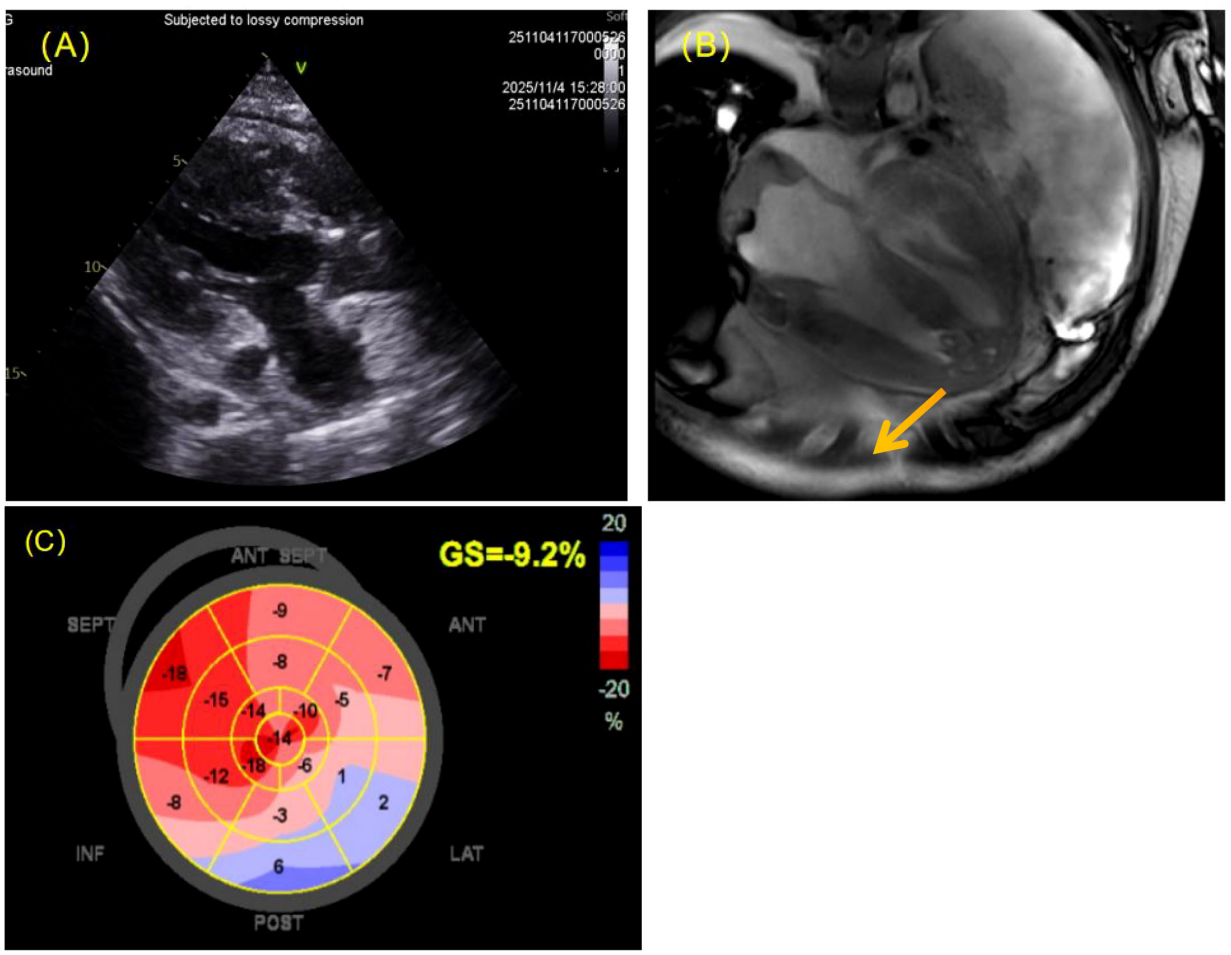
Cardiac imaging at presentation. **(A)** Transthoracic echocardiography reveals severe septal hypertrophy and right atrial enlargement. **(B)** CMR imaging shows diffuse myocardial hypertrophy and pericardial thickening. **(C)** Speckle-tracking echocardiography bull’s-eye plot demonstrates a markedly reduced global longitudinal strain (GLS) of −9.2%.

Transthoracic echocardiography demonstrated an LVEF of 47%, severe asymmetrical hypertrophy (interventricular septum 25 mm), right atrial enlargement, moderate tricuspid regurgitation, and reduced global systolic and diastolic function (**Figure 2A**). Speckle-tracking echocardiography revealed a markedly reduced global longitudinal strain (GLS) of −9.2%, indicating severely impaired global myocardial mechanical deformation (**Figure 2C**). CMR imaging revealed diffuse myocardial hypertrophy (maximum thickness 27 mm at the mid-anterior wall) involving both ventricles, right atrium, and interatrial septum, along with diffuse pericardial thickening and enhancement suggestive of inflammation and a right atrial mural thrombus (**Figure 2B**).

**Figure 3:**
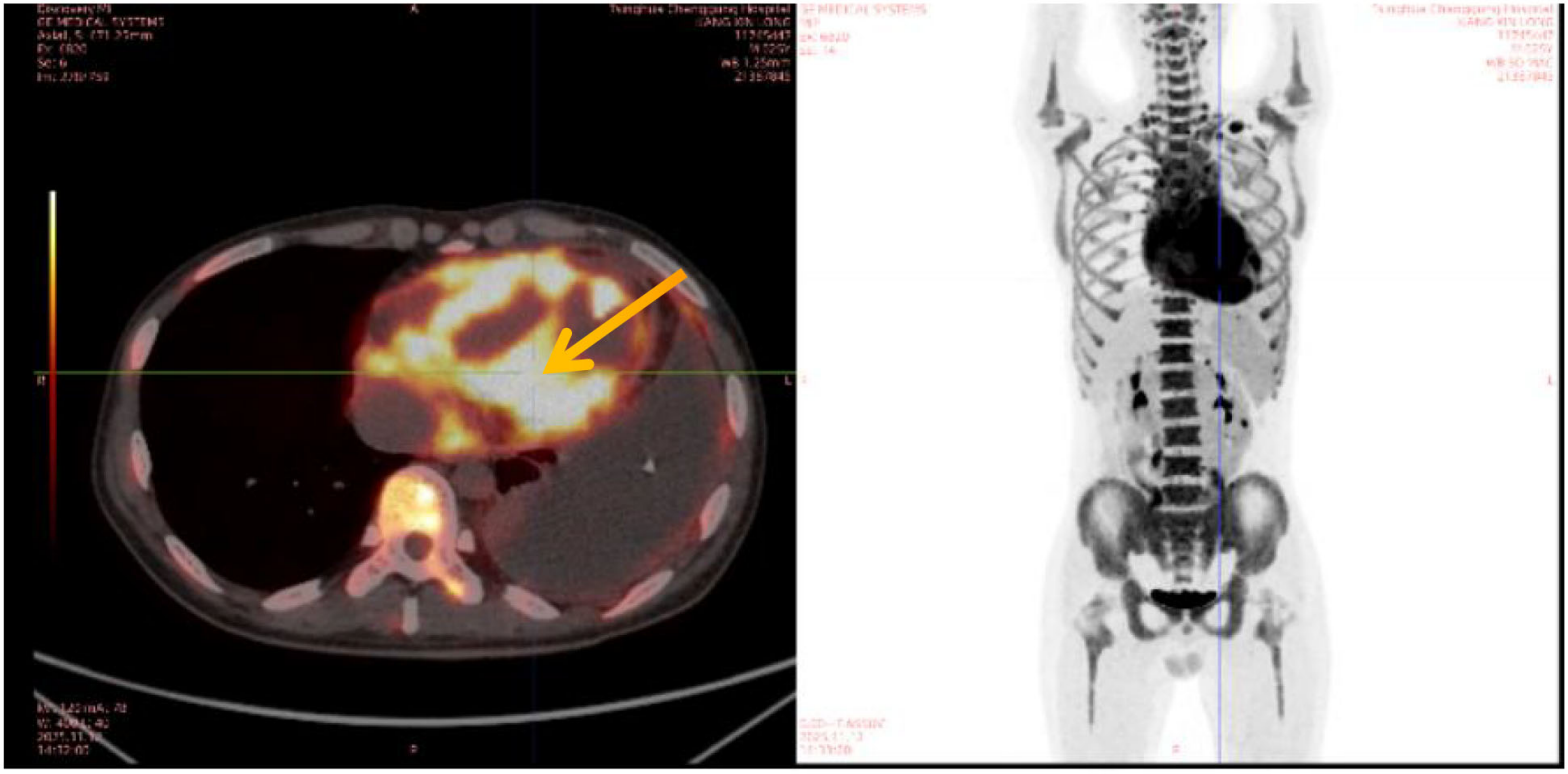
[^18^F]FDG PET–CT reveals diffuse myocardial and systemic FDG uptake. Intense myocardial FDG uptake (SUVmax 14.8) is accompanied by hypermetabolic activity in the pericardium, renal cortices, and multiple lymph node stations.

Because the cardiac findings could not explain the patient’s systemic symptoms, whole-body [^18^F]FDG PET–CT was performed. It demonstrated intense FDG uptake in the myocardium (SUVmax 14.8), pericardium and cardiac vascular spaces (SUVmax 6.9), bilateral renal cortical nodules (SUVmax 9.3), and multiple hypermetabolic lymph nodes in the supraclavicular, mediastinal, retroperitoneal, and left axillary regions (**Figure 3**). The findings were highly suspicious for a systemic hematological malignancy.

**Figure 4:**
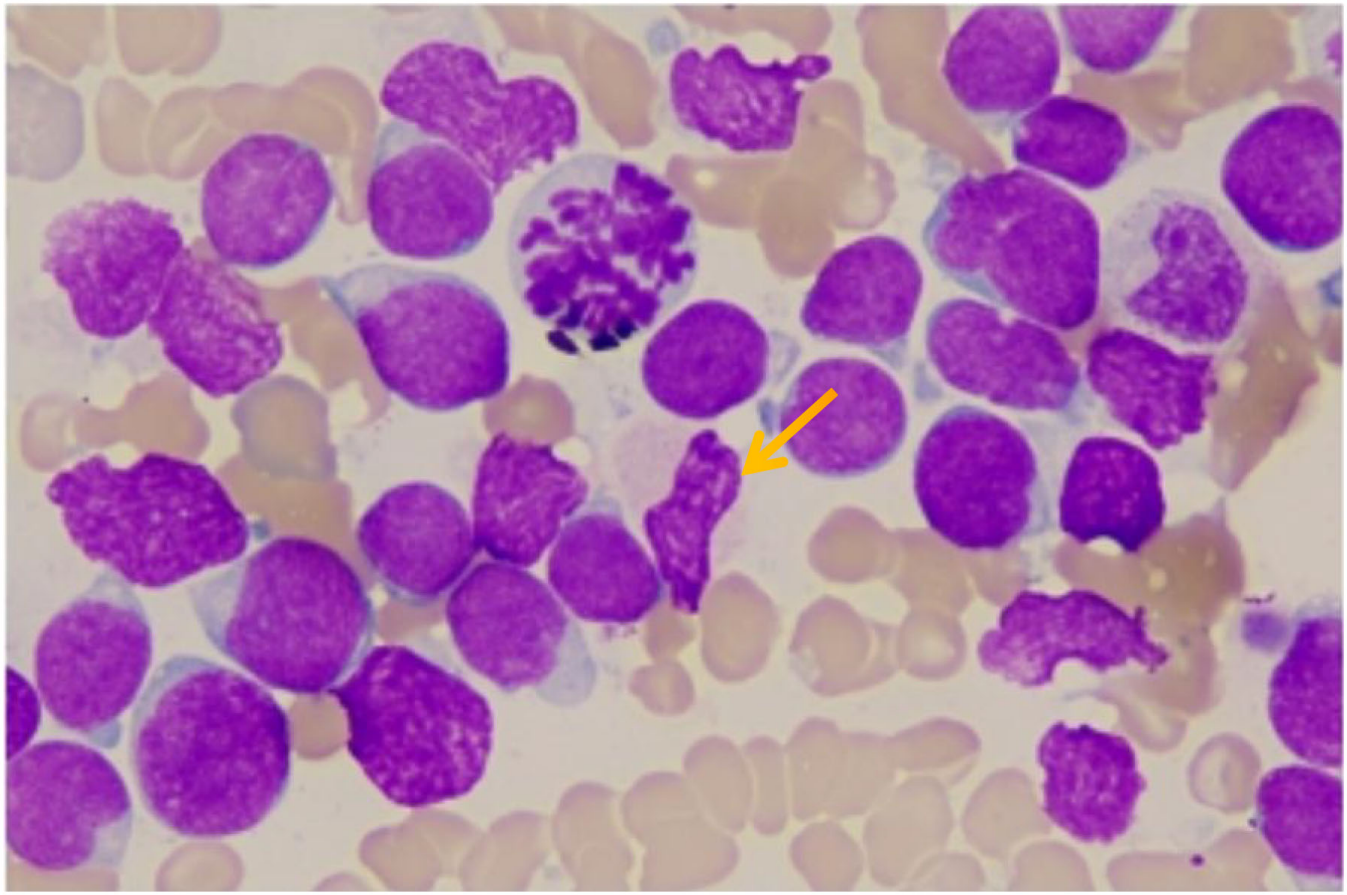
Bone marrow aspirate (Wright–Giemsa, ×1,000). The smear shows marked hypercellularity with numerous lymphoblasts (91%), characterized by a high nuclear-to-cytoplasmic ratio, fine chromatin, and prominent nucleoli.

Peripheral blood smear revealed 23% lymphoblasts, and bone marrow aspirate was hypercellular with 91% lymphoblasts (**Figure 4**). Immunophenotyping showed 69.04% abnormal immature T cells expressing CD2, CD3, CD5, CD99, CD38, CD33, cytoplasmic CD3, and TdT, while negative for CD4, CD8, CD1a, CD34, and CD117, consistent with T-ALL with myeloid co-expression. Genetic testing using next-generation sequencing (NGS) identified NRAS p.G12S and NOTCH1 p.Q2459X mutations, and WT1 and HOX11 fusion genes were positive. Bone marrow biopsy confirmed diffuse infiltration by T lymphoblasts (CD3^+^, CD5^+^, TdT^+^, Ki67 > 95%).

**Figure 5:**
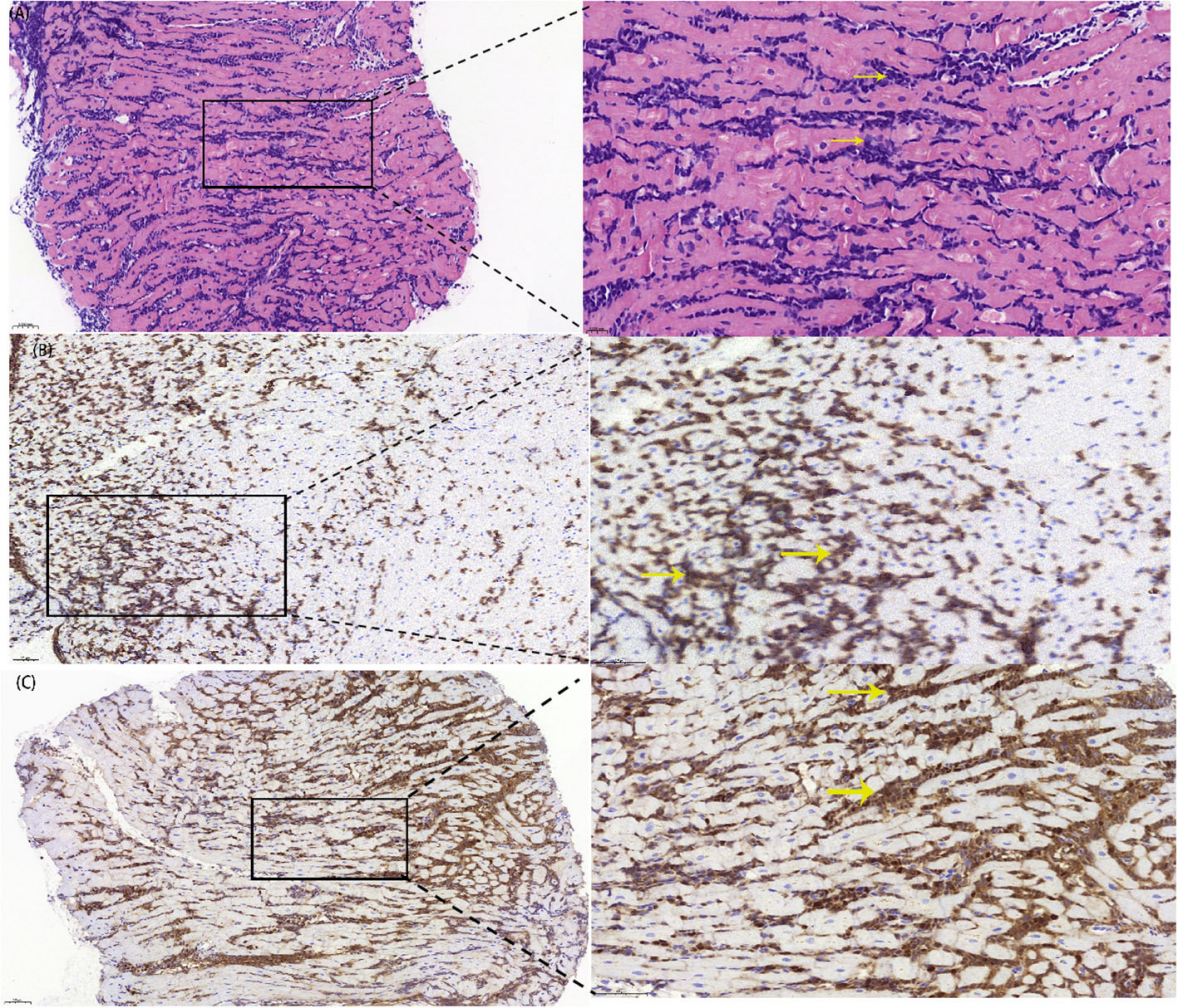
Histopathology of endomyocardial biopsy. **(A)** Hematoxylin and eosin (H&E) staining reveals diffuse infiltration of small lymphoid cells between myocytes. Left panel: 20× magnification; right panel: 40× magnification. **(B)** Immunohistochemistry for CD3 confirming the T-cell lineage of the infiltrating cells. Arrows indicate representative CD3-positive lymphoblasts. Left panel: ×20 magnification; right panel: ×40 magnification. **(C)** Immunohistochemistry for TdT positivity in the majority of the infiltrating lymphoid cells. Arrows indicate representative TdT-positive nuclei. Left panel: ×20 magnification; right panel: ×40 magnification.

Bone marrow examination subsequently confirmed T-ALL. However, the patient initially presented with severe cardiac dysfunction and systemic symptoms, and the cause of severe cardiomyopathy remained unclear. Therefore, endomyocardial biopsy was performed. Histopathology showed diffuse infiltration of small lymphoid cells in the myocardium. Immunohistochemistry revealed CD3^+^, CD5^+^, TdT^+^, CD8^+^ (subset), Ki67 95%, and EBER negative (**Figure 5**).

Based on the EGIL and WHO 2022 classification criteria, the patient was diagnosed with T-ALL with myeloid co-expression and initially mimicking non-obstructive hypertrophic cardiomyopathy. The biopsy provided histopathological proof that the cardiomyopathy was directly attributable to leukemic involvement. He started chemotherapy with the VDPL regimen (vincristine, daunorubicin, prednisone, L-asparaginase) on day 6 of admission, along with supportive care for heart failure and infection prophylaxis.

After the first cycle, he developed bone marrow suppression and bacteremia, which were managed with antibiotics and supportive care. Given the high-risk genetic features (WT1/HOX11 positivity and NRAS mutation) and the need for deep remission, subsequent cycles were tailored as consolidation/re-induction therapy, including venetoclax, chidamide, and azacitidine, followed by mitoxantrone and cytarabine in cycle 3, combined with intrathecal cytarabine for central nervous system prophylaxis. After three cycles of chemotherapy, the patient achieved complete remission with negative minimal residual disease by flow cytometry.

The patient was subsequently transferred to another hospital closer to his residence for continued follow-up. Telephone follow-up revealed that he remained in good clinical condition with substantial resolution of dyspnea and edema. However, detailed quantitative data could not be obtained. Nonetheless, the hematology team confirmed sustained complete remission without additional antileukemic therapy beyond the three cycles.

## Discussion

We report a rare case of T-cell acute lymphoblastic leukemia presenting with an HCM-like phenotype that was reversible after chemotherapy. This case provides several important clinical lessons and contributes to the growing literature on leukemic cardiac infiltration.

Leukemic cardiac infiltration is far more common at autopsy than appreciated clinically. Autopsy series have reported cardiac involvement in 20%–44% of patients with acute leukemia and lymphoid malignancies (3–5, 12, 22). A systematic review identified 30 cases of ALL presenting with cardiac manifestations as the first clinical sign, among which 8 were T-ALL, with the predominant presentation being massive pericardial effusion or cardiac tamponade (6). However, myocardial hypertrophy as the sole initial manifestation was extremely rare, accounting for only a minority of cases (6). To the best of our knowledge, our case is among the few documented instances of T-ALL presenting as isolated myocardial hypertrophy without concurrent pericardial effusion or cardiac mass. In our patient, initial echocardiography and CMR showed severe, asymmetrical left ventricular hypertrophy with reduced systolic function, features highly suggestive of HCM. However, several “red flags” pointed away from a primary cardiomyopathy. The patient had unexplained fever and significant weight loss of 20 kg over 3 months, along with markedly elevated inflammatory markers without an identifiable infectious source. His ventricular hypertrophy progressed rapidly from 21 to 27 mm over just weeks, and he showed no response to conventional heart failure therapy. There was no family history of HCM or sudden cardiac death. Furthermore, NT-proBNP was disproportionately elevated (2,103 pg/mL), while troponin was only mildly raised (19.4 ng/L), whereas in primary HCM or acute myocarditis, troponin elevation is typically more pronounced. Electrocardiographic abnormalities, although often non-specific, can also serve as an important red flag. In cancer patients without active cardiac symptoms, any new ECG change should raise suspicion for cardiac metastases, with ECG evidence of myocardial ischemia or injury demonstrating a high specificity of 96% for this diagnosis (21). These atypical features should prompt clinicians to consider secondary causes of myocardial hypertrophy, including infiltrative hematological malignancies.

While echocardiography and CMR delineate structural abnormalities, PET–CT was pivotal in our case to reveal the systemic nature of the disease, with intense FDG uptake in the myocardium (SUVmax 14.8), lymph nodes, and kidneys. The myocardial SUVmax of 14.8 is far higher than typically seen in HCM or cardiac amyloidosis (usually SUVmax <5–6) and should alert clinicians to a neoplastic or inflammatory process (6, 14). Beyond PET–CT, advanced CMR with parametric mapping has emerged as a valuable non-invasive tool to differentiate infiltrative from hypertrophic cardiomyopathy. For instance, a recent case of relapsed ALL presenting with biventricular hypertrophy demonstrated diffuse FDG uptake alongside elevated native T1 and extracellular volume (ECV) values, which were highly suggestive of diffuse cellular infiltration (13). In our patient, although parametric mapping was not available at the initial presentation, the complementary roles of PET–CT and CMR were crucial in raising the suspicion of a systemic hematological malignancy.

The pathogenesis of leukemic dissemination to the heart is not fully understood, but recent studies have provided important insights. Leukemic cells possess an innate ability for migration and tissue colonization, making acute leukemias an “ultimate example of aggressive metastasis” (15, 23). The chemokine CXCL12 has been shown to promote chromatin remodeling and nuclear deformation in T-ALL cells, facilitating their invasiveness through the endothelium and into peripheral tissues (24). The selective tropism of leukemic cells for the myocardium may be mediated by specific adhesion molecules and chemokine receptor axes, such as CXCR4−CXCL12 and VLA−4/VCAM−1, which are known to regulate leukemic cell homing to the bone marrow and extramedullary sites. Whether the heart provides a particularly favorable microenvironment for T lymphoblasts remains an open question that warrants further investigation, perhaps due to its rich vascularization and expression of certain chemokines (23).

The most striking aspect of our case is the marked improvement in cardiac structure and function after only three cycles of chemotherapy. Although follow-up imaging was performed at an outside hospital and the original images could not be obtained, telephone follow-up confirmed that the patient’s cardiac function had significantly improved. According to the patient’s report, subsequent echocardiography demonstrated reduction of septal hypertrophy and recovery of left ventricular ejection fraction. This reversibility underscores a critical pathophysiological point. The myocardial hypertrophy was not due to irreversible myocyte disarray or fibrosis as seen in primary HCM, but rather to interstitial infiltration by leukemic blasts that resolved with effective antileukemic therapy. A similar reversibility has been observed in other cases. A patient with ALL had focal left ventricular hypertrophy and systolic dysfunction that reversed rapidly after chemotherapy (14). Another case of T-ALL presenting with acute right ventricular failure due to extensive cardiac infiltration showed improvement after treatment (15). A 29-year-old male with ALL presenting as acute decompensated heart failure achieved normalization of cardiac function following chemotherapy (9). The consistent observation of reversibility across these reports supports the concept that leukemic cardiomyopathy is a potentially curable form of infiltrative heart disease, provided that timely diagnosis and appropriate chemotherapy are administered.

A noteworthy procedural point in our case is that endomyocardial biopsy (EMB) was performed after bone marrow had established the diagnosis of T-ALL, but before the cause of severe cardiac dysfunction could be attributed with certainty. While the bone marrow confirmed the systemic hematological malignancy, it could not distinguish whether the myocardial hypertrophy and systolic failure were due to direct leukemic infiltration, viral myocarditis, or stress-induced cardiomyopathy. EMB was therefore essential to confirm that the cardiac involvement was indeed leukemic in nature, which in turn provided the histopathological rationale for expecting reversibility with chemotherapy and justified the use of intensive cardiotoxic agents.

The core particularity of this case lies in cardiomyopathy as the initial clinical manifestation, with atypical symptoms related to hematological diseases, which is prone to clinical misdiagnosis and constitutes a rare clinical presentation type of acute lymphoblastic leukemia. A search of journals published in the past 10 years yielded 11 full-text accessible case reports of ALL complicated with cardiac infiltration (**Table 1**). Among these cases, five were T-cell type, five were B-cell type, and one was of unknown immunophenotype. The age of onset ranged from 4 to 43 years, predominantly involving young and middle-aged adults. Among these patients, the time interval from the diagnosis of ALL to the emergence of myocardial infiltration ranged from 0 to 30 months. This cohort exhibits a dismal prognosis, with approximately half of the patients ultimately dying from the disease.

**Table 1:** Summary of ALL cases complicated by cardiac infiltration.

Case	Author	Gender	Age (years)	ALL type	Time from diagnosis of ALL to onset of myocardial infiltration (months)	Clinical manifestations	Treatment	Outcomes
1	Kyongmin Sarah Beck (13), 2025	Female	43	Not mentioned	Not mentioned	Dyspnea and chest discomfort	Not mentioned	Not mentioned
2	Yigeng Cao (16), 2023	Female	43	B-cell type	30	Dyspnea and fatigue	Chemotherapy	Death
3	Wenhui Chu (25), 2023	Female	14	B-cell type	0	Numbness of limbs	Chemotherapy	Death
4	Kanako Niwa (26), 2022	Female	26	B-cell type	0	Cough and facial edema	Chemotherapy, allogeneic transplantation	Death
5	Meyer EJ (8), 2022	Female	4	T-cell type	0	Fatigue, fever, abdominal distension, rash	Chemotherapy	Not mentioned
6	Werhahn SM (15), 2020	Male	25	T-cell type	0	Shortness of breath, jugular vein distension, edema	Chemotherapy, allogeneic transplantation	Disease remission
7	Naman Sharma (27), 2019	Male	Not mentioned	B-cell type	12	Dyspnea	Chemotherapy	Not mentioned
8	James Nadel (10), 2018	Female	39	B-cell type	More than 6 months	Fever, fatigue, chest pain	TKI, ponatinib	Death
9	Dzmitry Fursevich (11), 2017	Male	39	T-cell type	18	Chest pain	Chemotherapy	Disease remission
10	Anna Baritussio (17), 2016	Male	38	T-cell type	More than 9 months	Rash, diplopia	Chemotherapy	Not mentioned
11	Stuart B. Prenner (14), 2016	Male	33	T-cell type	0	Fatigue and dry cough	Chemotherapy	Not mentioned

Based on our experience and a review of the literature (6, 10, 13, 15–20), we propose that endomyocardial biopsy should be considered in young patients with unexplained, rapidly progressive myocardial hypertrophy when accompanied by systemic symptoms, elevated inflammatory markers, and poor response to heart failure therapy, particularly in the absence of family history or genetic mutations. Although EMB is invasive and carries a small risk of perforation and arrhythmias, it provides definitive histopathological confirmation and can guide treatment decisions in diagnostically challenging cases. In our patient, the biopsy was performed safely and was critical in establishing the diagnosis after non-invasive workup was inconclusive. A diagnostic algorithm may proceed as follows. Initial evaluation should include echocardiography and CMR to assess hypertrophy and infiltrative patterns. If systemic symptoms or atypical features are present, PET–CT should be performed to evaluate extra-cardiac involvement and guide biopsy site selection. If PET–CT suggests diffuse infiltration, bone marrow examination and peripheral blood smear should be obtained. Only when non-invasive hematological workup is negative or inconclusive should endomyocardial biopsy be considered. Importantly, the presence of heart failure should not be considered a contraindication to chemotherapy; rather, prompt initiation of antileukemic therapy is essential to reverse cardiac dysfunction.

## Conclusion

T-ALL should be considered in the differential diagnosis of young patients presenting with unexplained, rapidly progressive myocardial hypertrophy, especially when accompanied by systemic symptoms, elevated inflammatory markers, and poor response to heart failure therapy. PET–CT demonstrating intense myocardial FDG uptake can provide a valuable clue to systemic malignancy. Early diagnosis via bone marrow examination and, if necessary, endomyocardial biopsy allows prompt initiation of chemotherapy, which can reverse life-threatening cardiac dysfunction. This case highlights that leukemic cardiomyopathy is a potentially curable form of infiltrative heart disease and underscores the importance of multidisciplinary collaboration between hematologists and cardiologists in managing such patients.

## Data Availability

The original contributions presented in the study are included in the article/supplementary material. Further inquiries can be directed to the corresponding author.
